# Extracts of *Tripterygium wilfordii* Hook F in the Treatment of Rheumatoid Arthritis: A Systemic Review and Meta-Analysis of Randomised Controlled Trials

**DOI:** 10.1155/2013/410793

**Published:** 2013-12-04

**Authors:** Yafei Liu, Shenghao Tu, Weina Gao, Yu Wang, Peilin Liu, Yonghong Hu, Hui Dong

**Affiliations:** Institute of Integrated Traditional Chinese and Western Medicine, Tongji Hospital, Tongji Medical College, Huazhong University of Science and Technology, 1095 Jiefang Avenue, Wuhan, Hubei 430030, China

## Abstract

Clinical trials have reported the effects of *Tripterygium wilfordii* Hook F (TwHF) extracts (TEs) in the treatment of rheumatoid arthritis (RA); however, the results have been inconsistent. This meta-analysis is aimed to assess the safety of TEs and their effects on the treatment of RA. Randomised controlled trials (RCTs) comparing the effects of TEs and placebo (PBO) or disease-modifying antirheumatic drugs (DMARDs) in patients with RA were included. Weighted mean differences (MDs) were calculated for net changes by employing fixed-effect or random-effects models. After filtering, ten RCTs (involving 733 participants) were included in this study. The methodological quality of these studies was generally low. Compared with DMARDs, TEs alone produced a mild increase in grip strength (GS) (*P* = 0.02; standard mean difference (SMD) = 0.81; 95% confidence interval (CI): 0.14 to 1.48). The most common adverse effects (AEs) of TEs were gastrointestinal discomfort, menstruation disorders, and amenorrhea. In conclusion, TEs, as a sort of “herbal DMARD,” could be as effective as synthetic DMARDs in the treatment of RA. However, the efficacy of TEs in treating RA should be further estimated with better designed, fully powered, confirmatory RCTs that apply the American College of Rheumatology (ACR) improvement criteria to evaluate their outcomes.

## 1. Introduction

Rheumatoid arthritis (RA) is an autoimmune disease of unknown aetiology that is characterised by pain, stiffness, and swelling of peripheral joints [1]. RA affects approximately 1% of the population worldwide [2]. Uncontrolled disease can result in progressive joint destruction, deformity, disability, and increased mortality. According to the guidelines proposed by the American College of Rheumatology (ACR) for the management of RA [3], nonsteroidal antiinflammatory drugs (NSAIDs) and disease-modifying antirheumatic drugs (DMARDs) are recommended to relieve joint damage. Recently, based on an evolving understanding of the pathogenic mechanisms of RA [4], specific targeted therapies (including inhibitors of tumour necrosis factor and other novel biological compounds) [5] have been introduced to interfere with the disease process in RA.

However, many patients discontinue the above treatments because of adverse events (AEs) [6] or poor clinical response to biological agents. Furthermore, biologics are unlikely to be of general benefit in the developing world because of the financial constraints [7], and the relatively high medical care costs for RA [8] restrict the application of these drugs in the developing world.

*Tripterygium wilfordii* Hook F (TwHF), commonly known as thunder god vine, is a member of the Celastraceae family. It is a perennial vine-like plant that is abundant in south China [9]. Anti-inflammatory and immunosuppressive compounds extracted from TwHF have been used for the treatment of a wide spectrum of autoimmune and inflammatory diseases, including RA [10, 11], ankylosing spondylitis [12, 13], and systemic lupus erythematosus [14]. Additionally, TwHF extracts (TEs) have been demonstrating beneficial effects on nephrotic syndrome [15], Crohn's disease [16], and solid tumours [17].

Among the approximately 380 secondary metabolites isolated from *Tripterygium *species, 95% are terpenoids [18]. Triptolide and tripdiolide, the ethyl acetate extract and chloroform-methanol extract [19], respectively, are the major components that account for the immunosuppressive effects of TwHF. It has been reported that these extracts exert better therapeutic effects and cause fewer AEs than other crude preparations. Therefore, these two preparations have been used most widely in China [10].

Meanwhile, many studies have been dedicated to elucidating the potential molecular mechanisms underlying the anti-inflammatory and immunosuppressive effects of TEs [18], including the inhibition of platelet activation [20], the induction of nitric oxide [21], and prostaglandin E_2_ [22] production. Based on studies both *in vitro* and *in vivo*, it is easy to speculate that TEs are likely to be types of herbal DMARDs, which differ from synthetic DMARDs.

While the extracts of TwHF have been most frequently used for a long time in treating RA, there exist a number of issues. In this regard, most of this clinical information comes from uncontrolled clinical trials or from retrospective reports, and few multicentre clinical trials have been performed to confirm the effects of TEs in the treatment of RA. In addition, the scientific evidence verifying that TEs are as effective as other conventional treatments in treating RA remains to be further validated. In terms of security, the safety of a long-term TE intake for chronic RA is uncertain. Given these uncertainties, it is necessary to assess the pertinent trials to systematically review the potential effects and safety of the long-term application of TEs in the treatment of RA.

## 2. Materials and Methods

To ensure the accuracy of our systemic review and meta-analysis, we designed and reported our results by employing a checklist of items that was as consistent as possible with the Preferred Reporting Items for Systemic Review and Meta-Analyses (PRISMA) statement.

### 2.1. Search Strategy

We searched the following digital databases to identify trials: PubMed, Embase, the Cochrane Library, and Clinical Trials.gov. In addition, we searched the Chinese databases, such as the CNKI Database, VIP Database, CBM Database, WanFang Database, and Chinese Clinical Trial Register. All of the databases were searched from their available dates of inception to the latest issue (January 2013).

Different search strategies were combined as follows. For the English databases, we used free text terms, such as “*Tripterygium wilfordii* Hook F,” “lei gong teng,” “thunder god vine,” or “yellow vine” (which are all alternative names in Chinese for *Tripterygium wilfordii *Hook F) and “rheumatoid arthritis,” or “RA.” For the Chinese databases, free text terms were used, such as “lei gong teng” or “huang teng” (which means* Tripterygium wilfordii* Hook F in Chinese) and “lei feng shi guan jie yan” (which means rheumatoid arthritis in Chinese). A filter for clinical trials was applied. To collect an adequate number of trials, the reference lists of relevant publications were also searched to identify additional studies.

### 2.2. Selection Criteria

Randomised controlled trials (RCTs) were included regardless of blinding, publication status, or language. Studies were selected for analysis if they satisfied the following criteria: (1) the subjects took extracts of TwHF alone or with other DMARDs for at least 4 weeks; (2) the study was an RCT with a parallel or crossover design; (3) TEs were used as an active treatment intervention; and (4) people enrolled were diagnosed with RA, according to the 1987 guidelines of the American Rheumatology Association [23].

“TEs,” in this review, mainly refer to the two root extracts of TwHF that have shown therapeutic promise, tripterygium glycosides tablets and tripterygium tablets. Therefore, studies using any TwHF-containing herbs or other herbal extracts were excluded. We also excluded case reports, reviews, retrospective studies, or studies without control groups. For obviously repeated studies, the authors of the reports were contacted to clarify any ambiguities. If the author could not be reached, the first published study was considered to be original. Studies were also excluded if the dose of TEs was not available. RCTs that lacked sufficient data to allow for the calculation of the net changes in outcomes and their variances from the baseline to the endpoint were also eliminated from our analysis. Two reviewers selected the articles independently. Based on the PRISMA requirements, a flow diagram of the study selection has been generated.

### 2.3. Data Extraction and Management

The relevant data was extracted by two independent reviewers, and divergences were resolved by consensus or were arbitrated by a third reviewer. The validated Jadad instrument was adopted to assess each study's quality independently [24]. The Jadad score included the following items: randomisation (0–2 points); double-blinding (0–2 points); and description of withdrawals and dropouts (0-1 point). Allocation concealment was estimated by the criteria adopted from Schulz et al. [25]. Studies with Jadad scores of no less than 3 were regarded as being of high quality.

The primary outcomes were tender joint count (TJC), swollen joint count (SJC), duration of morning stiffness (DMS), and grip strength (GS). The secondary outcomes consisted of rheumatoid factor (RF), erythrocyte sedimentation rate (ESR), and C-reactive protein (CRP). AEs were also collected from the studies. For the trials that applied a three-armed group design, the outcomes of the groups were extracted if they met the inclusion criteria and were excluded otherwise. In case of vagueness or absence in the articles of the outcomes, the authors were contacted and related data has been extracted by consensus if the authors were unavailable.

### 2.4. Data Synthesis and Analysis

The effects of TE intake on patients with RA were calculated as differences between the treatment groups and the no TE control group, by employing Review Manager meta-analysis software, version 5.2. To ensure the credibility of the results, the net changes in all of the outcomes were calculated as the mean differences (TEs minus control) in changes (endpoint minus baseline) for parallel trials. We calculated weighted mean differences (MDs) or standard mean differences (SMDs) and 95% confidence intervals (CIs) for continuous data. MDs were used if the outcomes were evaluated in the same manner among trials, while SMDs were used if the same outcomes were evaluated by adopting different approaches. Heterogeneity was evaluated via the chi-square test, the tau^2^ test, and Higgins *I* ^2^ test. A fixed-effect model was employed when the studies in the group were sufficiently alike (*P* > 0.10); otherwise, a random-effects model was used. A *Z* score was calculated to test the overall effect, with significance set at *P* < 0.05. Publication bias was detected by funnel plots, Egger's regression asymmetry test, and Begg's test when the number of included trials ≥5 (Stata software, version 12.0). We performed subgroup analyses to verify whether the use of TEs alone or with DMARDs had different effects on the outcomes.

To minimise the clinical heterogeneity, we performed three subgroup analyses: TEs compared with a placebo (PBO); TEs compared with DMARDs; and TEs with DMARDs compared with DMARDs alone.

## 3. Results

### 3.1. Study Selection

**Figure 1:**
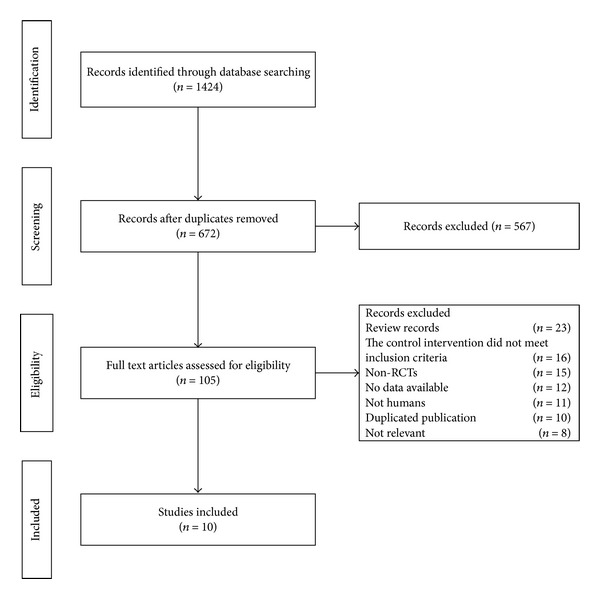
Process of searching for and screening studies.

The process of study selection is shown in Figure 1. According to the prespecified selection criteria defined in the Methods section, ten RCTs were included in the meta-analysis. In the PBO group, three studies were searched [26–28]. Two RCTs were crossover studies with two courses [26, 27]. One study was excluded for all of the outcomes were graphic representations [28]. Two studies [29, 30] that compared TEs with nonsteroidal anti-inflammatory drugs (NSAIDs) were also excluded from our review. The trial of Fu et al. [29] compared TEs with NSAIDs and physiotherapy, and in the other trial [30], the doses of NSAIDs changed during the treatment; thus, it was difficult to determine the effects of the intervention and the control groups. The characteristics of the studies are summarised in Table 1. Together, these studies included a total of 733 participants.

### 3.2. Study Descriptions

The included studies were published as full texts between 1988 and 2011. All of the RCTs originated in China, except for one [36]. Eight studies were published in Chinese, while two studies were in English [26, 36]. Nine studies were conducted as single-centre trials, and one study [36] was a multicentre trial.

### 3.3. Interventions and Controls

Two studies compared TEs with a PBO. Six studies randomised the participants to receive TEs alone versus a control of DMARDs. Two trials compared a cointervention of TEs and DMARDs (methotrexate, or sulfasalazine) with a control of DMARDs alone. There were three types of TE preparations applied in the included trials, consisting of tripterygium glycosides tablets, tripterygium tablets, and an unknown TE capsule. Different doses of TEs were used in these trials. The TE intake ranged from 0.01 g to 1.8 g per day. Except for two trials in which the TEs doses were not less than 0.18 g, the doses of TEs in most of the included trials were moderate (≤0.09 g). The total daily TE intake was divided into one to three doses. One trial [37] reduced the dose of TEs during the period of study when liver function abnormalities occurred. In the event of gastrointestinal intolerance, the protocol of one trial [36] allowed for a temporary dose reduction of 50%.

The duration of the interventions in the included studies also differed, ranging from four to twenty weeks. In the trial by Wang et al., the outcomes were detected at three time points: 20 weeks, 40 weeks, and 80 weeks [32]. Another trial had two time points: 4 weeks and 24 weeks [36]. To ensure homogeneity among the studies, we chose only 20 weeks and 4 weeks from the above two trials. The interventions lasted for four weeks in three trials [33, 36, 38], twelve weeks in five trials [26, 31, 34, 35, 37], and sixteen weeks in one trial [27]. Only one trial [32] reported that the patients had received TEs for twenty weeks.

### 3.4. Objectives and Outcomes

The majority of the outcomes of the study [36], such as TJC, SJC, ESR, CRP, were graphic representations, rather than outcomes reported in a table that allowed for the extraction of data for re-analysis. AEs were reported in eight trials. Eight trials performed treated-per-protocol analysis, and two [27, 36] performed intention-to-treat analysis which was generally interpreted as including all participants, regardless of the entry criteria, the treatment actually received, and ensuing withdrawal or deviation from the protocol.

### 3.5. Quality of the Included Studies

Compared with the four trials [26, 27, 36, 37] that were of high quality, most of the included trials were of low quality (Jadad score < 3) because of unclear randomisation, deficient allocation concealment, inadequate blinding, and undescribed withdrawals and dropouts. An adequate double blind was also performed in two of the four trials [26, 36]. Meanwhile, withdrawals and dropouts were described in four trials [26, 27, 36, 37].

### 3.6. Publication Bias

**Figure 2:**
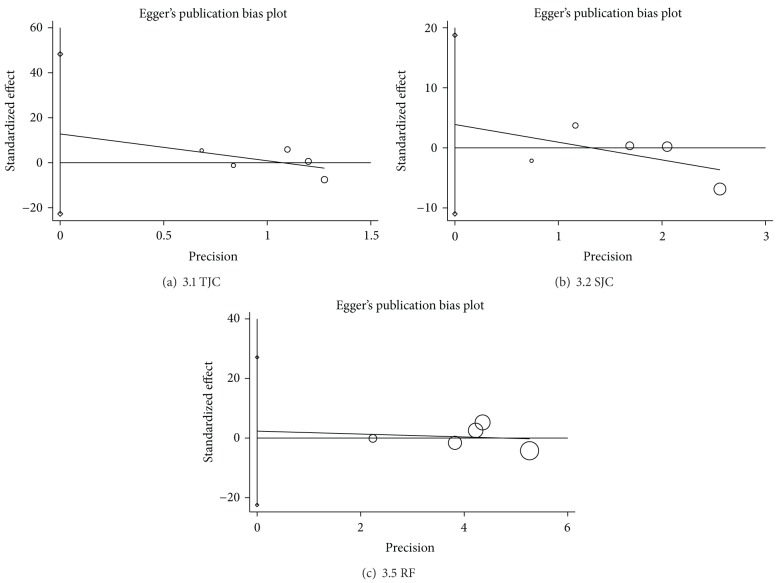
Publication bias in the included trials. Egger's linear regression test for detecting publication bias. TJC: tender joint count; SJC: swollen joint count; RF: rheumatoid factor. “◯” is a size graph symbol for the weight of each included study. The distance between two diamonds on the second vertical bar on the left represents the 95% CI for the intercept.

Egger's publication bias plots and Begg's test showed that there were no significant publication biases for three outcomes in which the numbers of the included trials were not less than 5. As shown in Figure 2, the calculated *P* values exceeded 0.05 in three outcomes among the studies (TJC, *P* = 0.335; SJC, *P* = 0.467; RF, *P* = 0.785), and the 95% CI for the intercept included zero. However, these results cannot be considered convincing because there were fewer than ten trials.

### 3.7. Effects of Interventions

#### 3.7.1. TEs Compared with a PBO

**Figure 3:**
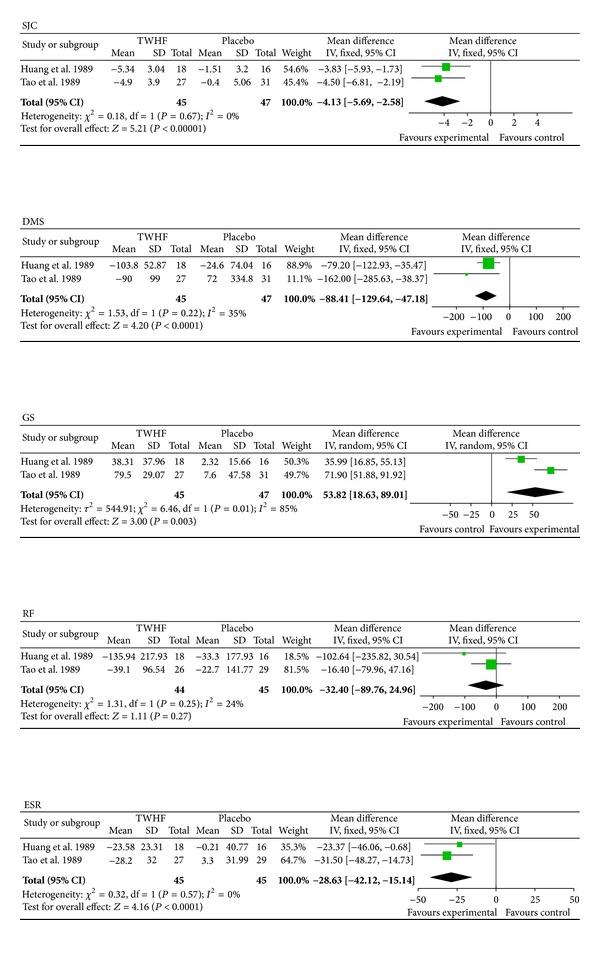
The first subgroup analyses comparing the effects of TEs and a PBO. Forest plots of TE treatment compared with a PBO. TEs: TwHF extracts; PBO: placebo; SJC: swollen joint count; DMS: duration of morning stiffness; GS: grip strength; RF: rheumatoid factor; ESR: erythrocyte sedimentation rate.

Two trials (involving 92 patients) compared the therapeutic effects of TEs and a PBO [26, 27]. The number of trial participants ranged from 16 to 31 with trial durations in the range of twelve to sixteen weeks. As shown in Figure 3, the statistical heterogeneity among the studies was found to be significant regarding the results for GS (*P* = 0.01). The pooled results indicated a significant difference between TE-treated group and the PBO group, aside from RF (*P* = 0.27; MD = −32.40; 95% CI: −89.76 to 24.96). The significant difference was identified between TEs and PBO in terms of the SJC (*P* < 0.00001; MD = −4.13; 95% CI: −5.69 to −2.58), DMS (*P* < 0.0001; MD = −88.41 min; 95% CI: −129.64 to −47.18), and ESR (*P* < 0.0001; MD = −28.63 mm/H; 95% CI: −42.12 to −15.14). A small but significant increase in GS (*P* = 0.003; MD = 53.82; 95% CI: 18.63 to 89.01) was also found.

#### 3.7.2. TEs Compared with DMARDs

**Figure 4:**
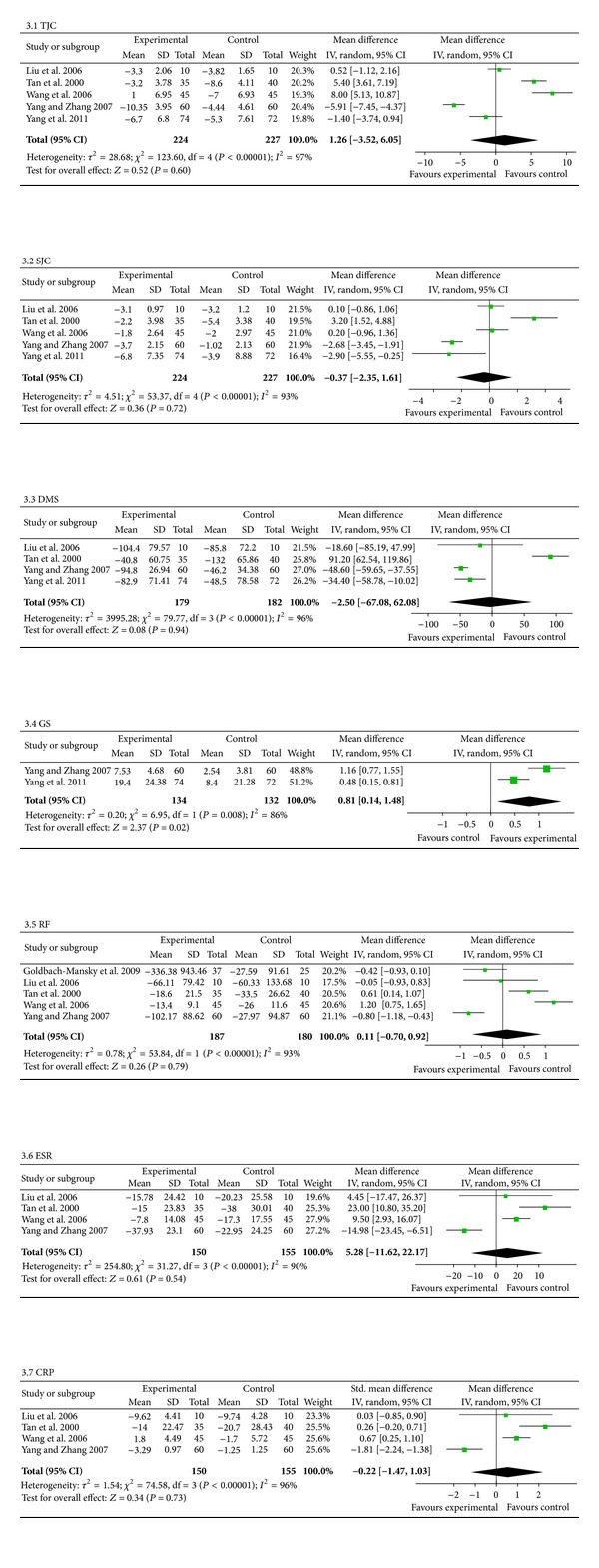
The second subgroup analyses comparing the effects of TEs with those of DMARDs. Forest plots comparing the effects of TE treatment with DMARDs. Note: TEs: TwHF extracts; DMARDs: disease-modifying antirheumatic drugs; TJC: tender joint count; SJC: swollen joint count; DMS: duration of morning stiffness; GS: grip strength; RF: rheumatoid factor; ESR: erythrocyte sedimentation rate; CRP: C-reactive protein.

Six trials (involving 513 patients) compared the therapeutic effects of TEs with those of DMARDs [31–36]. The number of trial participants ranged from 10 to 74, with the trial duration varying from four to twenty weeks. As illustrated in Figure 4, there was significant heterogeneity among the studies (all *P* < 0.10). Consequently, a random-effects model was employed to pool the results. The pooled results displayed no significant differences between TE-treated group and the DMARDs group, aside from GS (*P* = 0.02; SMD = 0.81; 95% CI: 0.14 to 1.48). However, no effects were found for TJC (*P* = 0.60; MD = 1.26; 95% CI: −3.52 to 6.05), SJC (*P* = 0.72; MD = −0.37; 95% CI: −2.35 to 1.61), DMS (*P* = 0.94; MD = −2.50 min; 95% CI: −67.08 to 62.08), RF (*P* = 0.79; SMD = 0.11; 95% CI: −0.70 to 0.92), ESR (*P* = 0.54; MD = 5.28 mm/H; 95% CI: −11.62 to 22.17), or CRP (*P* = 0.73; SMD = −0.22; 95% CI: −1.47 to 1.03). Only one trial [36] described the results, which were a 20% improvement in RA as defined by ACR (ACR 20) [39], ACR 50, and ACR 70, so we did not pool these results.

#### 3.7.3. TEs with DMARDs Compared with DMARDs Alone

**Figure 5:**
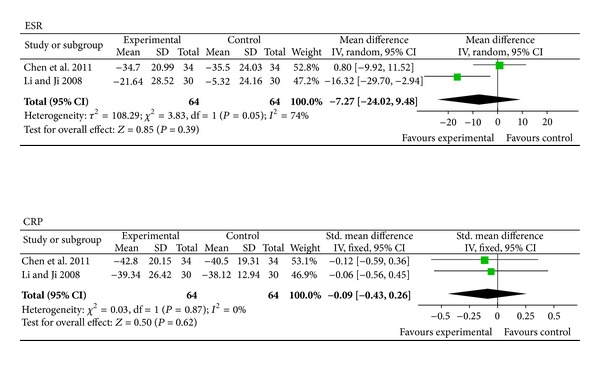
The third subgroup analyses comparing the effects of the coadministration of TEs and DMARDs with the effects of DMARDs alone. Forest plots comparing the effects of the coadministration of TEs and DMARDs with those of DMARDs alone. TEs: TwHF extracts; DMARDs: disease-modifying antirheumatic drugs; ESR: erythrocyte sedimentation rate; CRP: C-reactive protein.

Two trials (involving 128 patients) compared a combined therapy of TEs and DMARDs with DMARDs alone [37, 38]. The number of trial participants ranged from 30 to 34 with trial durations varying from four to twelve weeks. As shown in Figure 5, the statistical heterogeneity among the studies was found to be significant regarding the results for ESR (*P* = 0.05). The pooled results showed no significant differences between the two groups in terms of ESR (*P* = 0.39; MD = −7.27 mm/H; 95% CI: −24.02 to 9.48) or CRP (*P* = 0.62; SMD = −0.09; 95% CI: −0.43 to 0.26). Unfortunately, none of the included trials reported its results: ACR 20, ACR 50, or ACR 70.

### 3.8. AEs

Eight trials reported outcomes for AEs. Seven trials [26, 27, 31, 33, 34, 36, 37] reported mild to moderate gastrointestinal events in a few of the participants who received TEs. Menstruation disorders or amenorrhea was reported in six trials [26, 27, 31, 33, 36, 37] in the TE group. Three trials [27, 33, 37] reported mild liver function abnormalities in a few patients caused by the intake of TEs. In the trial by Chen et al. [37], study discontinuation occurred in one patient in each group, and another trial [36] reported that seventeen patients who received sulfasalazine and eight patients who received TEs discontinued the study because of AEs (*P* = 0.071). In addition, the same trial [36] reported that two patients became pregnant while receiving TEs or sulfasalazine, and mild prolongation of the corrected QT interval was seen on electrocardiography in patients receiving TEs.

## 4. Discussion

Although several systematic reviews and meta-analyses regarding the efficacy of TEs in the treatment of RA have been conducted, these systematic reviews achieved contradictory conclusions [40–43], which resulted from the differences of the search strategies, selection criteria, and data extraction and analysis, although all of these aspects have been recognised to some extent in these reviews. The meta-analysis published by Jiang et al. [43] (7 trials with 393 participants) performed two subgroup analyses: TEs versus PBO and TEs versus DMARDs. The systematic review published by Canter et al. [41] reported that TEs are associated with serious AEs, which render the risk-benefit analysis for TEs negative, and consequently, their application is not recommended. The most recent review, published by Cameron et al. [40] in the Cochrane Collaboration in 2011, could not pool its data due to differing interventions, comparisons, and outcomes. The Cochrane review concluded that TEs can reduce some RA symptoms; however, AEs can arise from oral use. We included ten trials and set three subgroups to minimize the heterogeneity, along with more new studies which made our systematic review differ from the previous ones.

Compared with a PBO, our results were consistent with those of Jiang et al. [43] in terms of SJC, RF, and ESR. In addition, TEs were found to be able to improve the DMS and GS. Although only two studies were included in this subgroup, the results showed that TEs were superior to PBO in improving joint function and reducing disease activity in RA.

Many of our results were consistent with the findings of Jiang et al. [43] between the TE-treated group and the DMARDs group, in terms of TJC, RF, and CRP. Unlike the previous review by Jiang et al. [43], there were no beneficial effects on SJC, DMS, or ESR in our review when comparing TEs with DMARDs. Furthermore, our review showed that the TE group had increased GS (SMD = 0.81) compared with the DMARDs group.

As shown in Figure 5, no beneficial effects on ESR and CRP were observed when the coadministration of TEs and DMARDs was compared with the administration of DMARDs alone. Although only two studies were included in this subgroup and two results were pooled, the analysis showed that TEs plus DMARDs had the same effects as those of two synthetic DMARDs alone in terms of lowering disease activity in RA. Additionally, the control groups, containing different efficient DMARDs, might have been responsible for the lack of intergroup differences in most of the endpoints.

The most common AEs with TEs were gastrointestinal discomfort, menstruation disorders, and amenorrhea, and they could be relieved with or without dose reductions. Due to different interventions, limited data, and the low quality of the included studies, the AEs were not ultimately combined. Identical to the synthetic DMARDs, the toxicity of TEs requires monitoring regularly to prevent AEs. Additionally, the doses of TEs should be controlled to avoid AEs.

However, some limitations of this meta-analysis should be noted. First, nine of the included trials were conducted in Chinese populations, which implies a high risk of selection bias. This fact could have influenced the applicability of TEs to populations of other ethnic origins. Second, most of the studies published in Chinese were of poor quality regarding their designs, reporting, and methodologies. Only one multicentre RCT was identified [36], which applied adequate randomisation, double-blinding, and allocation concealment in the included trials. As we know, if investigators, participants, and outcome assessors are not blinded, knowledge of group assignment can influence responses to an intervention [44]. Furthermore, inadequate allocation concealment resulted in exaggerated estimates of treatment effect [45]. Third, the limited number (from two to five) of the trials included in each subgroup obscured the positive evidence of TEs for RA. Fourth, the heterogeneity between the trials included in each subgroup was also significant, especially in the subgroup of TEs versus DMARDs. We believe that differences in the quality of the reports, intervention methods, doses, and durations of treatment were responsible for the heterogeneity. Different efficacy, applicability, and toxicity presented in different synthetic DMARDs also gave rise to heterogeneity. Finally, the most important criteria (ACR 20, ACR 50, ACR 70) were not reported in nearly any of the trials except for one study [36]. In view of this, we should carefully explain all of the conclusions due to the considerable methodological and clinical variety of the studies.

## 5. Conclusion

In summary, TEs alone or combined with DMARDs might not be inferior to DMARDs in the treatment of RA. Based on their bioactivity, TEs, which function as a type of “herbal DMARD,” appear to have the same effects as those of synthetic DMARDs. Meanwhile, the AEs of TEs should be assessed periodically, as with synthetic DMARDs. Considering the low methodological quality of the randomised trials, more RCTs are needed before we can recommend TEs to replace synthetic DMARDs or to be combined with them.

## Figures and Tables

**Table 1:** The characteristics of the included trials.

Author	Number of patients		Intervention and TwHF dose (g)		Duration (wks)	Outcomes
	Experimental	Control	Experimental	Control		
Tao et al. 1989 [26]	27	31	TEs (0.06)	PBO	12	SJC, DMS, GS, RF, ESR, AE
Huang et al. 1989 [27]	18	16	TEs (0.03)	PBO	16	SJC, DMS, GS, RF, ESR, AE
Tan et al. 2000 [31]	40	35	TEs (0.06)	MTX + PA	12	TJC, SJC, DMS, RF, ESR, CRP, AE
Wang et al. 2006 [32]	45	45	TEs (0.06)	MTX	20	TJC, SJC, RF, ESR, CRP, AE
Yang and Zhang 2007 [33]	60	60	TEs (0.06)	MTX	4	TJC, SJC, DMS, GS, RF, ESR, CRP, AE
Yang 2011 [34]	74	72	TEs (1.8)	MTX	12	TJC, SJC, DMS, GS, AE
Liu et al. 2006 [35]	10	10	TEs (0.09)	MTX	12	TJC, SJC, DMS, RF, ESR, CRP
Goldbach-Mansky et al. 2009 [36]	37	25	TEs (0.18)	SSZ	4	RF, AE
Chen et al. 2011 [37]	34	34	TEs (0.06), MTX	MTX + LEF	12	ESR, CRP, AE
Li and Ji 2008 [38]	30	30	TEs (0.01–0.02), SSZ	MTX + SSZ	4	ESR, CRP

Note: TEs: TwHF extracts; MTX: methotrexate; LEF: leflunomide; SSZ: sulfasalazine; PA: penicillamine; TJC: tender joint count; SJC: swollen joint count; DMS: duration of morning stiffness; GS: grip strength; RF: rheumatoid factor; ESR: erythrocyte sedimentation rate; CRP: C-reactive protein; AE: adverse effect.

## References

[1] Lee DM, Weinblatt ME (2001). Rheumatoid arthritis. *The Lancet*.

[2] Harris ED (1990). Rheumatoid arthritis: pathophysiology and implications for therapy. *New England Journal of Medicine*.

[3] Kwoh CK, Anderson LG, Greene JM (2002). Guidelines for the management of rheumatoid arthritis: 2002 update—American College of Rheumatology Subcommittee on Rheumatoid Arthritis Guidelines. *Arthritis and Rheumatism*.

[4] Firestein GS (2003). Evolving concepts of rheumatoid arthritis. *Nature*.

[5] Smolen JS, Aletaha D, Koeller M, Weisman MH, Emery P (2007). New therapies for treatment of rheumatoid arthritis. *The Lancet*.

[6] Bongartz T, Sutton AJ, Sweeting MJ, Buchan I, Matteson EL, Montori V (2006). Anti-TNF antibody therapy in rheumatoid arthritis and the risk of serious infections and malignancies: systematic review and meta-analysis of rare harmful effects in randomized controlled trials. *Journal of the American Medical Association*.

[7] Kalla AA, Tikly M (2003). Rheumatoid arthritis in the developing world. *Best Practice and Research: Clinical Rheumatology*.

[8] Yelin E, Wanke LA (1999). An assessment of the annual and long-term direct costs of rheumatoid arthritis: the impact of poor function and functional decline. *Arthritis and Rheumatism*.

[9] Tao X, Lipsky PE (2000). The Chinese anti-inflammatory and immunosuppressive herbal remedy *Tripterygium wilfordii* Hook F. *Rheumatic Disease Clinics of North America*.

[10] Tao X, Cush JJ, Garret M, Lipsky PE (2001). A phase I study of ethyl acetate extract of the Chinese antirheumatic herb *Tripterygium wilfordii* Hook F in rheumatoid arthritis. *Journal of Rheumatology*.

[11] Zhang W, Shi Q, Zhao LD (2010). The safety and effectiveness of a chloroform/methanol extract of *Tripterygium wilfordii* Hook F (T2) plus methotrexate in treating rheumatoid arthritis. *Journal of Clinical Rheumatology*.

[12] Ji W, Li J, Lin Y (2010). Report of 12 cases of ankylosing spondylitis patients treated with *Tripterygium wilfordii*. *Clinical Rheumatology*.

[13] Guo JL, Gao ZG, Zang AC, Bai RX (1986). Radix *Tripterygium wilfordii* Hook F in rheumatoid arthritis, ankylosing spondylitis and juvenile rheumatoid arthritis. *Chinese Medical Journal*.

[14] Patavino T, Brady DM (2001). Natural medicine and nutritional therapy as an alternative treatment in systemic lupus erythematosus. *Alternative Medicine Review*.

[15] Jiang X (1994). Clinical observations on the use of the Chinese herb *Tripterygium wilfordii* Hook for the treatment of nephrotic syndrome. *Pediatric Nephrology*.

[16] Ren J, Tao Q, Wang X, Wang Z, Li J (2007). Efficacy of T2 in active Crohn’s disease: a prospective study report. *Digestive Diseases and Sciences*.

[17] Yang S, Chen J, Guo Z (2003). Triptolide inhibits the growth and metastasis of solid tumors. *Molecular Cancer Therapeutics*.

[18] Brinker AM, Ma J, Lipsky PE, Raskin I (2007). Medicinal chemistry and pharmacology of genus *Tripterygium* (Celastraceae). *Phytochemistry*.

[19] Tao X, Cai JJ, Lipsky PE (1995). The identity of immunosuppressive components of the ethyl acetate extract and chloroform methanol extract (T2) of *Tripterygium wilfordii* Hook.F. *Journal of Pharmacology and Experimental Therapeutics*.

[20] Hu H, Straub A, Tian Z, Bassler N, Cheng J, Peter K (2009). Celastrol, a triterpene extracted from *Tripterygium wilfordii* Hook F, inhibits platelet activation. *Journal of Cardiovascular Pharmacology*.

[21] Wang B, Ma L, Tao X, Lipsky PE (2004). Triptolide, an active component of the Chinese herbal remedy *Tripterygium wilfordii* Hook F, inhibits production of nitric oxide by decreasing inducible nitric oxide synthase gene transcription. *Arthritis and Rheumatism*.

[22] Tao X, Schulze-Koops H, Ma L, Cai J, Mao Y, Lipsky PE (1998). Effects of *Tripterygium wilfordii* Hook F extracts on induction of cyclooxygenase 2 activity and prostaglandin E2 production. *Arthritis and Rheumatism*.

[23] Arnett FC, Edworthy SM, Bloch DA (1988). The American Rheumatism Association 1987 revised criteria for the classification of rheumatoid arthritis. *Arthritis and Rheumatism*.

[24] Jadad AR, Moore RA, Carroll D (1996). Assessing the quality of reports of randomized clinical trials: is blinding necessary?. *Controlled Clinical Trials*.

[25] Schulz KF, Chalmers L, Hayes RJ, Altman DG (1995). Empirical evidence of bias: dimensions of methodological quality associated with estimates of treatment effects in controlled trials. *Journal of the American Medical Association*.

[26] Tao XL, Sun Y, Dong Y (1989). A prospective, controlled, double-blind, cross-over study of *Tripterygium wilfodii* Hook F in treatment of rheumatoid arthritis. *Chinese Medical Journal*.

[27] Huang C, Wu C, Shen Z, Lin M Study on the treatment of rheumatoid arthritis with polyglycosides of *Tripterygium wilfordii* Hook F: randomized, controlled, crossover clinical trial.

[28] Tao X, Younger J, Fan FZ, Wang B, Lipsky PE (2002). Benefit of an extract of *Tripterygium wilfordii* Hook F in patients with rheumatoid arthritis: a double-blind, placebo-controlled study. *Arthritis and Rheumatism*.

[29] Fu J, Liang S, Ren L, Li W, Guo S (2001). The effect of tripterygium glycosides on plasma TNF*α* in patients with rheumatoid arthritis. *The Journal of Traditional Chinese Orthopedics and Traumatology*.

[30] Li N (2005). The effect of tripterygium glycosides on plasma TNF*α* and IL-6 in patients with rheumatoid arthritis. *Journal of Guangxi Medical University*.

[31] Tan Y, Wu C, You Y (2000). Clinical analysis of methotrexate combined with penicillamine in the treatment of rheumatoid arthritis. *China Journal of Modern Medicine*.

[32] Wang Y, Wu Q, Wei J, Shen H, Wang L, Zhu Q (2006). Total glucosides of paeony, methotrexate and *Tripterygium wilfordii* in the treatment of 150 cases of rheumatoid arthritis. *Journal of Zhengzhou University(Medical Sciences)*.

[33] Yang X, Zhang L (2007). Clinical observation of tripterygium for treatment of 60 cases of rheumatoid arthritis. *Chinese Journal of Traditional Medical Science and Technology*.

[34] Yang Z (2011). The clinic effect of *Tripterygium wilfordii* Hook F on rheumatoid arthritis. *China Pharmaceuticals*.

[35] Liu J, Li H, Chen X (2006). Effects of traditional Chinese medicine for invigorating spleen to resolve dampness and dredging collaterals on patients with rheumatoid arthritis and anemia. *Journal of Chinese Integrative Medicine*.

[36] Goldbach-Mansky R, Wilson M, Fleischmann R (2009). Comparison of *Tripterygium wilfordii* Hook F versus sulfasalazine in the treatment of rheumatoid arthritis: a randomized trial. *Annals of Internal Medicine*.

[37] Chen P, Zhu L, Zou X, Du H, Zhou Y, Gu X (2011). Tripterygium glucosides combined with methotrexate in treatment of rheumatoid arthritis: a randomised controlled trial. *Journal of Anhui Traditional Chinese Medical College*.

[38] Li N, Ji H (2008). *Tripterygium wilfordii* combines with sulfasalazine on the related indexes of active rheumatoid arthritis. *Journal of Liaoning University of Traditional Chinese Medicine*.

[39] Felson DT, Anderson JJ, Boers M (1995). American College of Rheumatology preliminary definition of improvement in rheumatoid arthritis. *Arthritis and Rheumatism*.

[40] Cameron M, Gagnier JJ, Chrubasik S (2011). Herbal therapy for treating rheumatoid arthritis. *Cochrane Database of Systematic Reviews*.

[41] Canter PH, Lee HS, Ernst E (2006). A systematic review of randomised clinical trials of *Tripterygium wilfordii* for rheumatoid arthritis. *Phytomedicine*.

[42] Little C, Parsons T (2000). Herbal therapy for treating rheumatoid arthritis. *Cochrane Database of Systematic Reviews*.

[43] Jiang Q, Cao W, Tang X, Juan J (2009). *Tripterygium wilfordii* extract for treating rheumatoid arthritis: systematic review. *China Journal of Chinese Materia Medica*.

[44] Schulz KF, Grimes DA (2002). Blinding in randomised trials: hiding who got what. *The Lancet*.

[45] Schulz KF, Grimes DA (2002). Allocation concealment in randomised trials: defending against deciphering. *The Lancet*.

